# Risk assessment of fungal materials

**DOI:** 10.1186/s40694-022-00134-x

**Published:** 2022-02-24

**Authors:** Jeroen G. van den Brandhof, Han A. B. Wösten

**Affiliations:** Microbiology, Department of Biology, Utrecht University, Padualaan 8, 3584 CH Utrecht, The Netherlands

**Keywords:** Biobased material, Mycelium material, Fungal material, Fungus, Mushroom forming fungus, Pathogenic fungus

## Abstract

Sustainable fungal materials have a high potential to replace non-sustainable materials such as those used for packaging or as an alternative for leather and textile. The properties of fungal materials depend on the type of fungus and substrate, the growth conditions and post-treatment of the material. So far, fungal materials are mainly made with species from the phylum Basidiomycota, selected for the mechanical and physical properties they provide. However, for mycelium materials to be implemented in society on a large scale, selection of fungal species should also be based on a risk assessment of the potential to be pathogenic, form mycotoxins, attract insects, or become an invasive species. Moreover, production processes should be standardized to ensure reproducibility and safety of the product.

## The need for sustainable materials

The world population is predicted to increase to 9.7 billion people in 2050 [1], thereby putting even more pressure on natural resources than is happening today. The materials used in industries are often non-sustainable and dominated by the linear economic model to make, use and dispose [2]. This linear approach is not sustainable and is already taken its toll on global ecosystems. Fungal materials have high potential to replace non-sustainable and/or polluting products and production processes. For instance, they could replace, at least in part, non-sustainable plastics, textiles, leather and construction materials. Petrochemical based plastics are widely used as packaging material. The amount of plastics produced globally amounts over 360 million metric tons per year [3] and has been growing annually by 8.4% in the period 1950–2015 [4]. Only 35% of the consumer plastics was recycled in Europe in 2020, 42% was incinerated for energy production, while 23% ended up in landfills [3]. Spreading of (micro-)plastics as litter or from landfills into the environment poses significant hazard to both terrestrial and marine ecosystems. Cotton production also impacts the environment by its high water demand and intense use of pesticides [5], while the leather industry uses chemicals such as chrome for tanning [6]. Finally, 4.2 giga tonnes of cement were produced worldwide in 2019, which coincided with a concerning amount of CO_2_ emissions [7]. Clearly, we need to shift towards a sustainable economy. The use of fungal materials may offer a fundamental change in our current way of manufacturing because these materials are produced from waste streams. Moreover, the fungal materials can be recycled at their end of life to make new materials, to use as feed or fertilizer, or to improve soil structure [8, 9]. In this review, we will discuss the risks of mycelium materials for workers, researchers, consumers and the natural environment due to the fungal life style and the production process of these materials. Based on this, we will make recommendations for their safe introduction on the market.

## Fungal materials

The growing interest in fungal materials during the past decade is illustrated by the increase in the number of publications, patent applications and start-ups in this field. Moreover, established companies like Adidas and Hermès have opened their doors for fungal materials [10]. Mycelium composites and pure fungal materials are distinguished. In the former case, a network of hyphae binds substrate particles together, whereas pure mycelium materials consists solely of fungal biomass. Wood degrading basidiomycetes are mainly used to produce these mycelium materials but species from other phyla have also been explored [11] (Table 1).

**Table 1:** Fungi described for use in materials in scientific publications. Publications were excluded in the case species were not defined or classified at genus level

Species (Phylum)	Edible (E)/medicinal (M)	Application (Reference)	Distribution	Habitat	Biome	Samples
*Abortiporus biennis* (B)#	‒	Pure mycelium [104]	Europe (33.3%) North America (30.4%) Asia (15.9%)	Forest (43.5%) Grassland (29%) Wetland (11.6%)	Soil (66.7%) Shoot (13%) Rhizosphere soil (10.1%)	69
*Agaricus bisporus* (B)	E*	Composite [105]; Nanopaper [21–23, 106–108]; Pure mycelium [109]	Europe (39.1%) Asia (31.5%) Africa (9.8%)	Grassland (30.4%) Cropland (23.9%) Forest (22.8%)	Soil (66.3%) Air (9.8%) Root (7.6%)	92
*Agrocybe aegerita* (B)	E*	Composite [110]	Europe (68.4%) South America (31.6%)	Cropland (68.4%) Forest (31.6%)	Soil (57.9%) Rhizosphere soil (31.6%) Root (10.5%)	19
*Allomyces arbusculus* (BL)	‒	Composite [11]; Nanopaper [107]	‒	‒	‒	‒
*Bjerkandera adusta* (B)#	‒	Composite [92]; Pure mycelium [104]	North America (44.4%) Europe (30.6%) Asia (22.3%)	Forest (78.1%) Anthropogenic (12.6%) Grassland (4.3%)	Root (45.6%) Air (19.4%) Deadwood (15.5%)	741
*Botrytis cinerea* (A)	‒	Composite [11]	Europe (59%) Asia (18.8%) North America (13.6%)	Forest (50.3%) Grassland (23.5%) Cropland (14.2%)	Soil (51.5%) Shoot (18.2%) Air (7.1%)	2994
*Ceriporia lacerata* (B)#	‒	Composite [111]	Asia (46.2%) North America (27.1%) South America (12.5%)	Forest (52.8%) Anthropogenic (15.8%) Aquatic (11.6%)	Soil (25.1%) Air (24.8%) Shoot (20.8%)	303
*Coprinellus micaceus* (B)	E	Composite [92]	Europe (72.3%) North America (13%) Asia (6.7%)	Forest (54.9%) Grassland (22.4%) Tundra (7.1%)	Soil (65.5%) Shoot (6.5%) Deadwood (6.3%)	1236
*Coprinopsis cinerea* (B)	E	Composite [99]	Europe (39.2%) Asia (37.5%) North America (22%)	Forest (36.1%) Cropland (30.3%) Grassland (22.8%)	Soil (55.3%) Air (15.9%) Shoot (14.1%)	347
*Daedaleopsis confragosa* (B)#	‒	Composite [89, 92]; Nanopaper [23]; Pure mycelium [104]	Europe (35.6%) Asia (31.8%) North America (28.8%)	Forest (38.6%) Grassland (24.2%) Anthropogenic (23.5%)	Air (30.3%) Soil (19%) Shoot (17.4%)	132
*Daedaleopsis tricolor* (B)#	‒	Pure mycelium [104]	‒	‒	‒	‒
*Flammulina velutipes* (B)	E*	Composite [105]	Asia (84%) Europe (12.5%) Australia (2.8%)	Aquatic (48.6%) Forest (22.9%) Anthropogenic (22.2%)	Forest (47.9%) Air (22.2%) Soil (18.1%)	144
*Fomes fomentarius* (B)#	M	Amadou [112–114]; Composite [115]; Pure mycelium [104]	Europe (93.1%) Asia (6.5%) Australia (0.4%)	Forest (84.9%) Grassland (8.6%) Anthropogenic (3.5%)	Air (26.7%) Soil (26.3%) Deadwood (19%)	232
*Fomitiporia mediterranea* (B)#	‒	Pure mycelium [104]	Asia, Europe	‒	‒	‒
*Fomitopsis iberica* (B)#	‒	Pure mycelium [104]	Europe	‒	‒	‒
*Fomitopsis pinicola* (B)#	M	Composite [91]; Pure mycelium [104]	Europe (95.7%) Asia (2.6%) North America (1.1%)	Forest (85.9%) Grassland (6.5%) Anthropogenic (4.8%)	Deadwood (31%) Air (22.9%) Soil (19.7%)	538
*Fusarium graminearum* (A)	‒	Pure mycelium [111]	Cosmopolitan*	‒	‒	‒
*Fusarium oxysporum* (A)	‒	Composite [11]	Europe (32.5%) Asia (25.1%) Australia (20.2%)	Forest (32.4%) Grassland (28.5%) Cropland (17%)	Soil (72.2%) Root (8.7%) Rhizosphere soil (6.9%)	6650
*Ganoderma applanatum* (B)#	M	Composite [116]	Widespread, records from tropics are likely *G. australe*	‒	‒	‒
*Ganoderma carnosum* (B)#	‒	Pure mycelium [104]	North America (63.3%) Europe (30%) Asia (6.7%)	Forest (58.3%) Wetland (25%) Grassland (10%)	Soil (78.3%) Rhizosphere soil (6.7%) Root (6.7%)	60
*Ganoderma curtisii* (B)#	M	Pure mycelium [27]	North America	‒	‒	‒
*Ganoderma lucidum* (B)#	M	3D-printing [19, 117]; Composite [11, 105, 118–123]; Pure mycelium [104, 124, 125]; Scaffold [126]	Asia (72.7%) North America (16.2%) Australia (8.4%)	Forest (89.5%) Woodland (3.6%) Cropland (1.8%)	Soil (82.9%) Rhizosphere soil (12.9%) Shoot (3%)	333
*Ganoderma mexicanum* (B)#	‒	Pure mycelium [27]	‒	‒	‒	‒
*Ganoderma resinaceum* (B)#	‒	Composite [18, 29, 89, 127, 128]; Pure mycelium [17]	Europe (100%); North Africa, Asia and North America	Cropland (91.7%) Grassland (8.3%)	Soil (50%) Rhizosphere soil (41.7%) Root (8.3%)	12
*Ganoderma sessile *(B)#	‒	Composite [129]	‒	‒	‒	‒
*Gloeophyllum odoratum* (B)#	‒	Composite [92]	Europe (100%); Circumpolar, North America rarely	Forest (61.1%) Anthropogenic (27.8%) Grassland (11.1%)	Air (61.1%) Deadwood (22.2%) Shoot (11.1%)	18
*Gloeophyllum sepiarium* (B)#	‒	Composite [91]	Europe (70.3%) North America (15.6%) Asia (10.9%)	Forest (56.3%) Grassland (15.6%) Anthropogenic (15.6%)	Air (40.6%) Soil (20.3%) Deadwood (12.5%)	64
*Grifola frondosa* (B)#	E*	Nanopaper [108]	Europe (60%) Asia (20%) North America (20%)	Forest (80%) Anthropogenic (20%)	Soil (60%) Root (20%) Air (20%)	5
*Hypsizygus marmoreus* (B)	E*	Nanopaper [108]	Asia (45.4%) North America (27.3%) Europe (27.3%)	Forest (77.3%) Grassland (4.6%) Wetland (4.6%)	Soil (63.6%) Rhizosphere soil (22.7%) Air (13.6%)	22
*Hypsizygus ulmarius* (B)	E*	Composite [11]	Asia (100%)	Forest (100%)	Rhizosphere soil (50%) Soil (50%)	6
*Irpex lacteus* (B)#	‒	Composite [130]; Pure mycelium [104]	North America (61.8%) Europe (27.3%) Pacific Ocean (7.3%)	Forest (72.7%) Anthropogenic (18.2%) Aquatic (7.3%)	Shoot (60%) Air (27.3%) Rhizosphere soil (7.3%)	55
*Irpiciporus pachyodon* (B)# syn. *Spongipellis pachyodon*	‒	Pure mycelium [104]	Temperate northern hemisphere	‒	‒	‒
*Kuehneromyces mutabilis* (B)	E*	Composite [92, 105]	Boreal and temperate North America	‒	‒	‒
*Laetiporus sulphureus* (B)#	E	Composite [91]	Europe (73.1%) Asia (17.1%) North America (9.8%)	Forest (41.5%) Anthropogenic (24.4%) Grassland (19.5%)	Air (51.2%) Soil (12.2%) Deadwood (9.8%)	41
*Lentinula edodes* (B)	E*	Composite [105]; Nanopaper [108]	Asia (100%) Australia	Forest (100%)	Soil (100%)	16
*Lentinus crinitus* (B)#	E	Pure mycelium [27]	North America (60%) South America (40%); Subtropical and tropical western hemisphere	Forest (100%)	Soil (80%) Root (20%)	5
*Lentinus velutinus* (B)#	E	Composite [131]	Subtropical and tropical regions	‒	‒	‒
*Lichtheimia corymbifera* (M)	‒	Composite [11]	Asia (100%)	Forest (40%) Grassland (40%) Cropland (20%)	Soil (100%)	5
*Megasporoporia minor* (B)#	‒	Composite [127]	Asia (44.4%) North America (22.2%) Europe (11.1%)	Forest (33.3%) Cropland (22.2%) Aquatic (22.2%)	Soil (44.4%) Shoot (33.3%) Rhizosphere soil (11.1%)	9
*Mucor genevensis* (M)	‒	Composite [11]; Nanopaper [107]	Europe (100%)	Forest (95.2%) Anthropogenic (4.8%)	Soil (45.2%) Litter (45.2%) Deadwood (9.7%)	62
*Mucor mucedo* (M)	‒	Pure mycelium [111]	Europe (51.5%) North America (36.4%) Asia (12.1%)	Forest (78.8%) Shrubland (9.1%) Anthropogenic (6.1%)	Soil (36.4%) Rhizosphere soil (33.3%) Litter (18.2%)	33
*Neofavolus alveolares* (B)# syn. *Polyporus alveolaris*	M	Pure mycelium [104]	Asia (45.8%) Europe (29.2%) North America (20.8%)	Anthropogenic (41.7%) Forest (41.7%) Tundra (8.3%)	Air (33.3%) Shoot (25%) Soil (20.8%)	57
*Oxyporus latemarginatus* (B)#	‒	Composite [127]	Cosmopolitan	‒	‒	‒
*Panus conchatus* (B)#	‒	Pure mycelium [27]	Europe (52.4%) Asia (33.3%) North America (9.5%)	Forest (42.9%) Anthropogenic (33.3%) Aquatic (19.1%)	Air (52.4%) Shoot (28.6%) Soil (9.5%)	21
*Phaeolus schweinitzii* (B)#	‒	Composite [91]	Europe (58.3%) North America (41.7%); Temperate northern hemisphere; Asia	Forest (36.1%) Tundra (33.3%) Wetland (13.9%)	Soil (83.3%) Air (8.3%) Rhizosphere soil (5.6%)	36
*Phellinus ellipsoideus* (B)#	‒	Amadou [113, 132]	Asia (100%)	Forest (100%)	Soil (100%)	3
*Phycomyces blakesleeanus* (M)	‒	Pure mycelium [111]	‒	‒	‒	‒
*Phytophthora cinnamomi* (O)	‒	Composite [11]	Primarily southern hemisphere	‒	‒	‒
*Piptoporus betulinus* (B)#	‒	Amadou [112]; Composite [91]	Europe (98.2%) North America (1.2%) South America (0.6%)	Forest (77.3%) Grassland (13.2%) Anthropogenic (4.8%)	Air (77.3%) Soil (14.4%) Shoot (4.2%)	167
*Pleurotus albidus* (B)	E	Composite [131]	South America (100%)	Forest (100%)	(Soil 100%)	4
*Pleurotus citrinopileatus* (B)	E*	Composite [11, 118]	Asia (100%)	Anthropogenic (42.9%) Cropland (42.9%) Forest (14.2%)	Air (42.9%) Root (42.9%) Soil (14.2%)	7
*Pleurotus cornucopiae* (B)	E*	Composite [11]	Europe (66.7%) Asia (33.3%)	Grassland (33.3%) Cropland (33.3%) Forest (33.3%)	Soil (33.3%) Air (33.3%) Shoot (33.3%)	3
*Pleurotus djamor* (B)	E*	Composite [11, 99]	North America (80%) South America (13.3%) Africa (6.7%)	Forest (86.6%) Mangrove (6.7%) Cropland (6.7%)	Soil (80%) Root (20%)	15
*Pleurotus eryngii* (B)	E*	Composite [11, 118, 133]; Nanopaper [108]	Africa (50%) Europe (50%)	Forest (50%) Cropland (50%)	Deadwood (50%) Soil (50%)	2
*Pleurotus ostreatus* (B)	E*	3D-printing [19]; Composite [11, 12, 91, 105, 110, 118, 123, 133–139]; Nanopaper [140]; Pure mycelium [27, 125]; Scaffold [126]	Asia (50.7%) Europe (33.8%) Australia (7.4%)	Forest (49.3%) Anthropogenic (33.1%) Cropland (11%)	Soil (34.6%) Air (36%) Shoot (17.7%)	136
*Pleurotus pulmonarius* (B)	E*	Composite [11, 110]	North America (58.8%) Europe (23.5%) Australia (5.9%)	Forest (76.5%) Grassland (5.9%) Anthropogenic (5.9%)	Shoot (47.1%) Soil (17.6%) Air (17.6%)	17
*Pleurotus salmoneostramineus* (B)	E*	Composite [110]	‒	‒	‒	‒
*Polyporus arcularius* (B)#	E	Composite [91]	Temperate northern hemisphere; Cosmopolitan	‒	‒	‒
*Polyporus brumalis* (B)#	M	Composite [11, 141]	Temperate northern hemisphere; Circumpolar	‒	‒	‒
*Pycnoporus sanguineus* (B)#	M	Composite [14, 131, 133]	Americas, Africa, India	‒	‒	‒
*Rhizomucor miehei* (M)	‒	Pure mycelium [111]	Asia (90%) Australia (10%); Widespread	Anthropogenic (50%) Cropland (40%) Forest (10%)	Air (50%) Soil (50%)	10
*Rhizopus oryzae* (M)	E*	Pure mycelium [111, 142]	Cosmopolitan	‒	‒	‒
*Saksenaea vasiformis* (M)	‒	Composite [11]	Africa (50%) North America (25%) Europe (25%)	Grassland (50%) Forest (25%) Shrubland (25%)	Soil (100%)	4
*Schizophyllum commune* (B)	E	Composite [12, 92]; Pure mycelium [25, 26, 92, 143]	Asia (54.4%) Europe (15%) North America (14.9%)	Forest (42.3%) Aquatic (16.5%) Anthropogenic (12%)	Soil (37.4%) Shoot (21.1%) Air (13.9%)	754
*Stereum hirsutum* (B)	‒	Pure mycelium [104]	Europe (66.2%) North America (17.3%) Asia (13.5%)	Forest (70.7%) Anthropogenic (11.1%) Grassland (8.3%)	Deadwood (37.3%) Air (21.1%) Shoot (17.3%)	577
*Stropharia rugosoannulata* (B)	E	Composite [11]	Europe (91.7%) Pacific Ocean (8.3%)	Grassland (66.7%) Forest (25%) Aquatic (8.3%)	Soil (41.7%) Air (25%) Root + rhizosphere soil (16.7%)	12
*Terana caerulea* (B)	‒	Pure mycelium [104]	Europe (53.3%) Asia (33.3%) Africa (6.7%)	Anthropogenic (53.3%) Grassland (26.7%) Forest (6.7%)	Air (46.7%) Dust (26.7%) Soil (13.3%)	15
*Trametes betulina* (B)# syn. *Lenzites betulinus*	‒	Composite [92]; Pure mycelium [104]	Europe (67.9%) North America (18.2%) Asia (13.2%)	Forest (73.6%) Anthropogenic (16.4%) Grassland (8.2%)	Deadwood (36.5%) Air (30.2%) Shoot (12.6%)	159
*Trametes gallica* (B)# syn. *Coriolopsis gallica*	‒	Pure mycelium [104]	Europe (89.4%) Atlantic Ocean (5.3%) North America (5.3%)	Grassland (38.6%) Anthropogenic (31.6%) Forest (15.8%)	Air (70.2%) Soil (10.5%) Shoot (8.8%)	57
*Trametes hirsuta* (B)#	M	Composite [92, 139]; Pure mycelium [104]	Europe (68.3%) Asia (14.5%) North America (8.1%)	Forest (62.9%) Grassland (16.1%) Anthropogenic (11.3%)	Air (44.1%) Soil (23.7%) Shoot (9.7%)	186
*Trametes multicolor* (B)#	‒	Composite [92, 129]; Nanopaper [144]	‒	‒	‒	‒
*Trametes pubescens* (B)#	‒	Composite [91]	Temperate northern hemisphere	‒	‒	‒
*Trametes suaveolens* (B)#	M	Composite [91]; Pure mycelium [104]	Asia (100%); Circumboreal; Europe	Forest (61.5%) Anthropogenic (38.5%)	Soil (61.5%) Air (23.1%) Dust (15.4%)	13
*Trametes trogii* (B)# syn. *Coriolopsis trogii*	‒	Pure mycelium [104]	Asia (76%) Europe (20.7%) Australia (1.7%)	Anthropogenic (62%) Cropland (11.6%) Forest (11.6%)	Air (63.6%) Soil (19%) Dust (9.9%)	121
*Trametes versicolor* (B)#	M	Composite [11, 18, 89, 92, 127, 129, 141, 145, 146]; Nanopaper [107]	Europe (66.9%) North America (13.7%) Asia (12.2%)	Forest (70.6%) Anthropogenic (10.3%) Grassland (7.7%)	Deadwood (37.2%) Air (19.5%) Soil (19.5%)	1029
*Trichaptum abietinum* (B)#	‒	Composite [91]	Europe (85.5%) North America (9.5%) Asia (3.2%)	Forest (78.2%) Anthropogenic (7.9%) Grassland (7.6%)	Deadwood (28.4%) Air (27.6%) Soil (19.2%)	380
*Trichoderma asperellum* (A)	‒	Composite [105]	Europe (30.5%) Asia (30.4%) North America (15.6%)	Forest (55.4%) Cropland (10.3%) Grassland (9.9%)	Soil (78%) Sediment (5.3%) Root (4.3%)	1696
*Tricholoma terreum* (B)	‒	Nanopaper [147]	Europe (50.2%) Asia (28.3%) Australia (21.2%)	Forest (60.8%) Grassland (24.8%) Cropland (6.4%)	Soil (88.1%) Air (2.9%) Rhizopsphere soil (2.9%)	311
*Xylaria hypoxylon* (A)	‒	Composite [92]	North America (86.1%) Europe (8.1%) South America (5.4%)	Forest (96.1%) Tundra (1.7%) Cropland (1%)	Shoot (76.3%) Soil (15.4%) Root (6.6%)	410

(A) Ascomycota; (B) Basidiomycota; (BL) Blastocladiomycota; (M) Mycoromycota; (O) Opisthosporidia; (#) Polypore fungi based on [27, 30, 59, 60]; (syn.) Taxonomic synonyms based on [59, 60]. Use as edible or medicinal fungus is based on [100] and indicated with (*) when grown commercially, while distribution is based on [59, 68, 102, 103]. Habitat and biome are based on [102]. *Samples* refer to the number of samples per species listed in the database [102]. *Biome* is the ecosystem where samples have been collected. *Shoot* includes all plant parts aboveground either dead or alive. Percentages indicate the fraction of samples per species per *distribution*, *habitat* or *biome*

Composite mycelium materials are usually made by growing the fungus in a substrate, often a low-cost organic waste stream. During colonization, the mycelium acts as a glue that binds the substrate particles together. The first step in the process of making a composite material is the selection of the species and substrate. Commonly used substrates are for instance hemp shives, different types of straw, and sawdust. The pasteurized or sterilized substrate is inoculated with colonized substrate from a previous batch or with spawn (i.e. a highly nutritious substrate like grains that is colonized by the fungus). Another approach is the use of mycelium that has been blended in water or medium. The inoculated substrate is grown in a mould for several days up to a month depending on species, substrate, sample dimension and growth conditions [12, 13]. Alternatively, the substrate is pre-grown in for instance bags and then transferred to a mould. At a certain moment, the colonized substrate is removed from the mould and dried or growth is prolonged to mature the material followed by drying. The drying process is essential to metabolically inactivate or to kill the fungus. Drying temperature can range from room temperature to 100 °C, while drying time varies between a few hours to several days [12, 14]. Drying at room temperature will normally inactivate but not kill the fungus. For instance, dried material of *Ganoderma* sp. could regrow a year after it had been dried at ambient temperature [15]. In contrast, a temperature of ≥ 60 °C will normally kill the fungus. The resulting mycelium composite has foam-like properties with a density of 60–300 kg/m^3^. It can be used for insulation because of its inherent low thermal conductivity and high acoustic absorption [13]. Mycelium composite materials absorbs 70–75% of the sound at frequencies < 1500 Hz [16] and have a thermal conductivity of 0.04–0.08 W/(m K) [13], both of which are similar to traditional insulation materials. Dried mycelium composite can be (heat) pressed to obtain materials with cork- and wood-like mechanical properties [12]. These pressed composites can be coated with resin and used as flooring [13], while the use of pressed mycelium composite as building materials is also being explored.

Besides inactivating (e.g. by drying at ambient temperature) or killing (e.g. by heat-drying) the fungus can also be maintained metabolically active to create a biocomputational material. For instance, a living composite made with *Ganoderma resinaceum* responds to pressure by changing its electrical activity [17]. Moreover, mycelium can be maintained active to enable production of large size mycelium composites, for instance to make mycelium connections between mycelium panels [15, 18, 19].

Pure mycelium materials are made by using liquid- or solid-state fermentation. Growing pure mycelium in a solid-state fermentation is similar to the way mycelium composites are produced. In the case of pure mycelium, however, the fungal skin that develops at the substrate-air interface is harvested. A CO_2_ concentration of 5–7% by volume and a temperature of 30–35 °C is used to inhibit mushroom development of *Ganoderma* sp. and to stimulate aerial hyphae formation, resulting in a thick felty skin [20]. Pressing the skin during and after growth is common practice to obtain a desired density [20]. Mushrooms resulting from solid-state fermentation can also be used to make pure mycelium materials. Mycelium films can be obtained by casting and drying blended mushrooms. Mycelium can also be processed before casting. For instance, white button mushrooms (*Agaricus bisporus*) has been used to make chitin–glucan based nanopaper [21–24].

Static and dynamic conditions can be used for liquid-state fermentation. Growth conditions such as agitation, pH, oxygen, temperature, light, medium composition and amount of inoculum are being optimized for each species. After the growth phase the fungal biomass is harvested. In the case of liquid static cultures, a sheet of mycelium is harvested that has formed at the water–air interface [25, 26]. In contrast, total mycelium is separated from the spent medium by filtration or centrifugation from liquid shaken or bioreactor cultures resulting in a “pulp” of biomass. The biomass can be directly casted and dried or first homogenized before casting and drying. The mycelium films resulting from static or liquid fermentations can be processed to modify properties. For instance, treatment with the plasticizer glycerol (≥ 8%) results in elastomer-like materials that are more hydrophilic than untreated material [27].

Pure mycelium materials can be used as a foam, a cellular scaffold or as a meat alternative [20]. Moreover, the material can be physically and/or chemically processed to manufacture leather-like materials. The use as textiles is also being explored. Like mycelium composites, one may wish to keep the pure mycelium metabolically active in the final product to use it for instance as smart wearables [28]. By measuring electrical activity it was shown that pure *Ganoderma resinaceum* mycelium responds to mechanical and optical stimulation [29]. This opens up a completely new range of fungal material applications, such as sensors and biocomputers.

## The fungal life style

The Kingdom Fungi consists of nine phyla, of which the Ascomycota and Basidiomycota represent most species [30]. According to the Catalogue of Life the current number of identified fungal species exceeds 146.000 [31] but the total number of species is predicted to range between 1.5 and 12 million [32, 33]. Fungi play a vital role in most ecosystems by interacting with other living organisms such as plants, animals, and algae [32]. A fraction of the fungi can establish mutual beneficial interactions with for instance plants (mycorrhizae) and algae and/or cyanobacteria (lichens). On the other hand, fungi can be pathogens of for instance animals, plants and other fungi. A distinction is made between opportunistic and classical pathogens that infect weakened or healthy individuals, respectively. Fungal pathogens may prevent species to become too dominant in ecosystems but can have devastating effects as well. For instance, members of the genus *Armillaria* (Basidiomycota) are aggressive pathogens causing root disease that affect trees and shrubs worldwide [32, 34]. Even more impactful, around 600 fungal species can infect humans. The far majority of these fungi are opportunistic fungi that infect individuals with a compromised immune system [35]. For instance, *Pleurotus ostreatus* (oyster mushroom) (Basidiomycota) and *Saccharomyces cerevisiae* (Ascomycota) that are commonly used as food or to produce food, respectively, are opportunistic pathogens that can cause allergies and serious infections, albeit at low frequency [36, 37]. Apart from infections, fungi can also give rise to disease by causing allergies and by production of mycotoxins. In addition, fungi play an important role in nature in nutrient cycling by degrading organic waste streams such as plant material. In fact, fungi are the main degraders of lignin in wood [30]. Saprotrophic fungi that degrade wood are classified as white-, brown- and soft-rot fungi. White-rot fungi (mainly Basidiomycota) degrade cellulose, hemicellulose and lignin, whereas brown-rot (Basidiomycota) degrade cellulose and hemicellulose, but do not depolymerize lignin [30, 32, 38, 39]. Soft-rot is dominated by soil-inhabiting Ascomycota that break down cellulose and hemicellulose and lignin as well, albeit at a much lower rate [32].

Fungi are typically opportunistic by adopting their life style when (a)biotic conditions change. Endophytes adopt the various fungal life styles and illustrate fungal opportunism. Endophytes reside in plants, either actively colonizing the host or simply being present in a dormant state [40, 41]. Endophytes can be harmless or beneficial, for instance by producing alkaloids that protect against grazing [32, 40] or by promoting plant growth and fruit production [40, 42]. However, endophytes can also be pathogens or switch from a mutualistic to a parasitic mode of growth due to changes in the (a)biotic environment of the fungus [40, 41].

## Dispersion of fungi in nature

Fungi reproduce by producing (a)sexual spores [43]. For instance, asexual conidia are formed by specialized structures in ascomycetes, while asexual chlamydospores are formed by vegetative hyphae of Ascomycota and Basidiomycota [32, 44, 45]. Moreover, Ascomycota and Basidiomycota form sexual asco- and basidio-spores. Part of the spores (mainly sexual spores) are considered hardly motile by being immobilized in the fruiting body. Other spores (both sexual and asexual) will be dispersed by water, air or by other vectors such as animals. Typically, every cubic meter of air contains 1000 to 10,000 spores [43]. Spores can be very stress resistant. For instance, *Paecilomyces variotii* (Ascomycota) forms the most heat resistant conidia reported to date with a decimal reduction time of more than 20 min at 60 °C [46]. Ascospores can be even more heat resistant. For instance, those of *Talaromyces flavus* (Ascomycota) have a decimal reduction time exceeding 5 min at 91 °C [47]. Spores can also be highly resistant to drought, salt, radiation and oxidative stress conditions. For example, conidia of *Aspergillus niger* (Ascomycota) are extremely resistant against X-ray, cosmic and UV-C radiation thereby likely to survive space travel [48]. Spores germinate when conditions are favourable. Notably, only part of the conidia will germinate when exposed to such conditions [49]. Only 20% of the conidia of *A. niger* germinate in the presence of 50 mM glucose. The majority of these spores thus remain in their stress resistant resting state. This provides a bet hedging strategy to prevent for instance that all germlings die when temperature exceeds the cardinal temperature of 47 °C during daytime [49].

Less common is the formation of a specialized structure known as sclerotium [32, 50] that is extremely stress-resistant and able to survive for years [32, 51]. Sclerotia are aggregates of hyphae with a tough outer layer of thick pigmented hyphae [32, 50] and their formation is triggered by stressful conditions [51]. Sclerotia of *L. mylittae* can even germinate and form a basidiocarp without external water [32].

The dispersion of fungi can have a huge impact on food production, human health and biodiversity. For instance, they cause extinction of amphibians [52]. A single basidiocarp is able to release 1 billion spores a day [53] but human activities such as trade, transport and travel are also important ways of spreading spores [32, 52]. For instance, transport of plant material and presence of the fungus on shoes, clothes and equipment are important factors of spreading *Fusarium* TR4 (Ascomycota), thereby causing wilting of Cavendish bananas throughout the world [54].

## Risk assessment

### Pathogenic fungi

Some of the fungal species that are used or have been proposed to use for mycelium materials have been reported to be pathogens of humans, animals and/or plants (Table 2). However, none of these animal or human pathogens are considered classical pathogens. The majority of the species that are used to make mycelium materials are white-rot basidiomycetes belonging to the subphylum Agaricomycotina [30]. Some of these wood degrading fungi can incidentally cause disease in human as opportunistic human pathogens. Exposure to high numbers of basidiospores can cause respiratory problems as observed in growers of the oyster mushroom *P. ostreatus* [36, 55]. Moreover, agaricomycetes can infect humans with a compromised immune system. For instance, a total of 71 *S. commune* infections (mainly broncho-pulmonary mycosis and sinusitis) have been reported worldwide until 2013 [56]. This number of infections should be related to the 57 reported cases of fungemia (until 2003) caused by *S. cerevisae* that is widely used in baking and brewing and as a probiotic [37] and the 150 million severe cases of fungal infections each year, of which 1.7 million patients die [57]. The opportunistic nature of agaricomycetes makes that one can work with these fungi at the lowest biosafety level during their production, although regulations may differ between countries.

**Table 2:** Pathogenic species used or proposed to make mycelium materials

Species	Description (Reference)
*Abortiporus biennis*	Heart rot [68]
*Agrocybe aegerita*	Necrotrophic parasite [148]
Bjerkandera adusta*	Pathogen on different species of trees and reported as a human pathogen [149]; Trunk rot [103]
Botrytis cinerea*	Plant pathogen [11, 32]; *Botrytis* rot also known as grey mould causing stem rot, seedling wilt and fruit rot on various plant families [68]; Necrotroph, can infect more than 200 plant species [58]
*Daedaleopsis confragosa*	Trunk rot [103]; Necrotrophic parasite [148]
*Flammulina velutipes*	Causes white rot and may be harmful to host plants [65]; Xylem rot on various woody plants [68]; Trunk rot [103]
Fomes fomentarius*	Found on living and dead hardwoods [59], and can cause mottled rot and trunk rot [68]; Xylem endophyte considered to be pathogenic [40]; Necrotrophic parasite [148]; Trunk rot [103]
*Fomitiporia mediterranea*	Associated with trunk diseases such as esca in grapevines [64, 65, 68]
Fomitopsis pinicola*	Necrotrophic parasite [148]; Heart rot [59, 103] on living conifers and black cherry and decay in timber [68]
Fusarium graminearum*	Plant pathogen, including for corn, wheat, rice and various plant families [68]; Highly destructive pathogen of all cereal species [58]; Plant pathogen and health risk for humans and animals [97]
Fusarium oxysporum*	Plant pathogen [11, 32] and human pathogen [68]; Soil-borne pathogen that causes vascular wilt on a wide range of plants [58]
*Ganoderma applanatum*	Causing heart and butt rot, pathogen in perennial crops and natural forests in India [62]; Butt rot [103]; Necrotrophic parasite [148]; Causing several types of rot in trees of multiple plant families [68]
*Ganoderma lucidum*	Pathogenicity on hardwoods [66]; Pathogen in perennial crops and natural forests in India [62]; Necrotrophic parasite [148]; Butt rot and lethal root rot in trees of multiple plant families [68]
*Ganoderma resinaceum*	Pathogen in perennial crops and natural forests in India [62]; Necrotrophic parasite [148]; Heart rot on various trees [68]
*Grifola frondosa*	Found on roots of living trees, hardwoods and conifers [59], and can cause butt rot [68]; Root pathogen [150]; Necrotrophic parasite [148]
*Hypsizygus ulmarius*	Necrotrophic parasite [148]
*Irpiciporus pachyodon*	Canker and white rot (but not decay of heartwood) [68]; Necrotrophic parasite [148]
Irpex lacteus*	Mostly strictly saprotrophic, but can cause cankers, decay and mortality of weak trees [68]
*Laetiporus sulphurous*	Pathogen causing heart rot [150]; Necrotrophic parasite [148]; Rot and hollowing in living hardwoods, especially *Quercus* [68], and conifers [59]; Trunk rot [103]
Lichtheimia corymbifera*	Human [151] and animal [152] pathogen
*Mucor genevensis*	Fruit rot in *Carica papaya* [68]
*Mucor mucedo*	Rot in multiple plant families [68]
*Neofavolus alveolaris*	Necrotrophic parasite [148]
Oxyporus latemarginatus*	Pathogenicity on hardwood hosts [66]
*Phaeolus schweinitzii*	Necrotrophic parasite [148]; Root and butt rot [103] on gymnosperms [68]; Found on roots of living trees [59]
*Phytophthora cinnamomi*	Plant pathogen [11]; Serious pathogen of hardwood forests and various crop species [68]; pathogen for oak trees [98]
*Piptoporus betulinus*	Heart rot in *Betula* [103] being latent present [150]; Necrotrophic parasite [148]
Pleurotus ostreatus*	Pathogenicity on trees and nematodes [149]; Necrotrophic parasite [148]; Heart rot [103]; Can cause respiratory problems when cultivated [36, 55]
*Pleurotus cornucopiae*	Necrotrophic parasite [148]
*Pleurotus eryngii*	Necrotrophic parasite [148]
*Pleurotus pulmonarius*	Causes distinct white rots in dead and living wood [65]; Necrotrophic parasite [148]
Pycnoporus sanguineus*	Plant pathogen [153]
*Rhizomucor miehei*	Human pathogen that can cause mycotic diseases [154]
Rhizopus oryzae*	Root rot, fruit rot, chlorosis and wilting on various plants [68]; Human pathogen [155]
*Saksenaea vasiformis*	Human and animal pathogen [152]
Schizophyllum commune*	Xylem rot [103]; Weak pathogen on grapevine [65]; Plant pathogen invading living wound tissue and can cause rot [156]; Infection in humans [56, 151]
Stereum hirsutum*	Necrosis, associated with esca and heart rot [68]
Trametes trogii*	Necrotrophic parasite [148]
Trametes hirsuta*	Wound pathogen mainly on older grapevines [65]; Opportunistic pathogen infecting through wounds [68]
*Trametes suaveolens*	Necrotrophic parasite [148]; Heart rot [103]
Trametes versicolor*	Pathogen on apple and other trees [149]; Opportunistic pathogen [68]

*Also part of Table 3

Plants are more prone to fungal infections than animals. Quite some fungi listed in Table 1 have been reported to be pathogenic for plants (Table 2). In fact, *Botrytis cinerea, Phytophthora cinnamomi* and *Fusarium* spp. are listed as regulated non-quarantine organisms in Europe, while *Fusarium oxysporum* f. sp. albedinis is even listed as a quarantine organism. Moreover, *B. cinerea*, *F. oxysporum* and *Fusarium graminearum* that are listed in Table 2 are considered to be in the top 5 of fungal pathogens based on scientific or economic importance [58]. In all other cases, fungi listed in Table 2 are not mentioned by the European Food Safety Authority. This implies that one can work with these fungi at the lowest biosafety level during material production. Yet, some of them are considered serious pathogens. Most of the 43 polypores listed in Table 1 are strictly saprotrophic, some grow and on dead wood in living trees, and a small number can invade and kill living wood [59, 60]. The polypores of *Ganoderma* that are often used to make mycelium material (Table 1) are considered serious pathogens for plantations and natural forests especially in Southeast Asia, causing tremendous economic loss [45, 61, 62]. For instance, *Ganoderma boninense* causes a destructive disease in palm plantations known as basal stem rot [45, 61]. In the past this disease was mainly found on older plants, but nowadays even young plants are affected [61]. Stem rot caused by *Ganoderma* species is also a disease for coconut palms [63]. White-rots (both polypore and gilled fungi) have been described as pathogens of grapevines [64, 65]. The main cause of white rot in grapevine is *Fomitiporia mediterranea*, however, other opportunistic species have also been described as a causative agent [64]. For instance, *Flammulina velutipes*, *P. ostreatus*, *S. commune* and *Trametes hirsuta* have been observed on grapevine in Europe, generally on weakened plants that have other diseases or wounds [65]. Another study identified white-rot fungi, also used for mycelium materials, on living fruit and nut trees at the West Coast of the United States [66] (Table 2). Occurrence was in most cases associated with wounded trees. Among others, *Oxyporus*, *Ganoderma* and *Trametes* species were often found on cherry trees, whereas species belonging to *Pleurotus* and *Laetiporus* were more common on walnut trees. In addition, white- and brown-rots can cause decay of wooden structures [67]. In particular, *Gloeophyllum sepiarium* causes decay in houses [59] and wooden objects such as railroad and utility poles [68].

A fraction of the fungi listed in Table 1 have been reported as endophytic fungi (Table 3). Endophytes should be used with caution, especially when introduced from a different continent since co-evolution between host and the fungus did not take place. Hence, resistance has not evolved which can make hosts highly susceptible [40]. It has been described that a change in lifestyle from endophytic to pathogenic can be caused by a mutation in a single locus [41]. The fact that disease is often only detected when sporocarps are formed complicates risk management. Furthermore, little is known how the intra-species genetic variation affects the life style of the fungus [32, 33]. This genetic variation can be high. For instance, a diversity of 0.2 has been found within synonymous sites of *S. commune* [69].

**Table 3:** Endophytes used or proposed to make mycelium materials

Species	Description (Reference)
Bjerkandera adusta*	Endophyte [157] in healthy trees [158]
Botrytis cinerea*	Endophyte [157]
*Ceriporia lacerata*	Endophyte [157]
*Coprinellus micaceus*	Endophyte [157]
*Coprinopsis cinerea*	Endophyte [157]
Fomes fomentarius*	Endophyte [40, 157] in healthy beech trees [44, 150]
Fomitopsis pinicola*	Endophyte [157]
Fusarium graminearum*	Endophyte [157]
Fusarium oxysporum*	Endophyte [157]
*Ganoderma carnosum*	Endophyte [157]
Irpex lacteus*	Endophyte [157]
Lichtheimia corymbifera*	Endophyte [157]
Oxyporus latemarginatus*	Endophyte [157] isolated from red peppers [80]
Pleurotus ostreatus*	Endophyte [157]
*Polyporus arcularius*	Endophyte [157]
Pycnoporus sanguineus*	Endophyte [157]
Rhizopus oryzae*	Endophyte [157]
Schizophyllum commune*	Endophyte [157]
Stereum hirsutum*	Endophyte [157]
Trametes gallica*	Endophyte [157]
Trametes hirsuta*	Endophyte [157]
Trametes versicolor*	Endophyte [157] in grapevine in southern Europe [65]
*Trichoderma asperellum*	Endophyte [98, 157]
*Xylaria hypoxylon*	Endophyte [157]

*Also part of Table 2

### Mycotoxin production

Some fungi listed in Table 1 produce mycotoxins, some of which can even be used as a biological weapon. Isolates of *F. oxysporum* [70], *F. graminearum* [58] and *Aspergillus* [71] are known to produce a variety of mycotoxins. Basidiomycetes also have the ability to produce toxins, leading to hundreds of deaths every year [72]. For instance, the white-rot fungus *Galerina marginata* is considered highly poisonous due to its ability to produce amatoxin [73, 74]. Mycotoxin levels are strictly monitored in food, but it may be relevant for fungal materials as well depending on their application. For instance, mycelium materials may be in direct contact with human skin when used as leather-like materials. It has been shown that mycotoxins can penetrate the skin [75] and therefore use of fungi producing mycotoxins should be avoided. Of importance, apart from *F. oxysporum* and *F. graminearum* none of the fungi listed in Table 1 are known to produce mycotoxins.

### Impact on the biotic environment

Fungi and insects are abundant in nature and they have evolved different interactions [76]. Insects benefit from fungi as food source, mechanical protection and antimicrobial defence. Fungi benefit from insects in a similar way, while insects also serve as a vector for fungal spore dispersal [76]. Fungi are known to produce complex mixtures of volatiles. The composition of volatile compounds can vary depending on growth conditions [77, 78] and developmental stage [79]. Hundreds of volatiles have been identified, including alcohols, aldehydes, esters, phenols and ketones [80, 81]. Volatiles are synthesized as by-products of metabolism and can have a protective or attractive role in interaction with animals [75, 76]. A well-known compound is the alcohol 1-octen-3-ol [80, 81], which can act both as attractant and repellent depending on the fungus-insect interaction [82]. Female flies are attracted by volatiles to lay eggs on the fruiting body to provide larvae with fungal tissue as a food source [32, 83]. In some cases, this is mutually beneficial when dispersal of fungal propagules by the insect takes place [76, 83]. These interactions can also result in the attraction of generalist predator insects to prey on fungus-insects [84]. Ants can also be attracted to mushrooms [85–87]. For instance, the ant species *Euprenolepis procera* is a specialist in harvesting of and living on fruiting bodies [85, 86].

A range of volatile compounds have been identified in species listed in Table 1. Most publications are about volatile compounds from (fresh) basidiocarps [80, 84], while few discuss volatile compounds of vegetative mycelium. The mycelial volatile compounds of the commonly used species *Trametes versicolor* and *P. ostreatus* are listed in Tables 4 and 5, respectively. An important aspect for insect herbivores to recognize host plants is the perception of the whole blend of volatiles. Therefore, testing individual compounds may not always be representative for insect behaviour [88]. Studying the natural effects of fungal volatile compounds has similar challenges [81]. Together, fungal materials may attract certain insects, while repelling others. When fungal materials are widely implemented in society they could impact insect biodiversity. Coating of mycelium materials is an effective way to prevent such effects. For instance, coating of pressed mycelium composites with a mixture of guayule resin and vegetable oil improves resistance against termites [89].

**Table 4:** Volatile compounds of *T. versicolor* when grown on beech wood [159] or potato dextrose [159, 160] in the absence of mushroom formation

Compound (Class)	Beech wood	Potato dextrose	Interaction (Reference)
1,2-Dimethylcyclopropane (H)	+		
2-Methylbutane (H)	+		
Isopropyl alcohol (Alc)	+	+	
2-Methylpentane (H)	+		
2-Butanol (Alc)	+		
3-Methylfuran (F)	+		
Dimethyl carbonate (Es)	+		
Methyl propionate (Es)	+		
3-Methyl-2-butanone (K)	+	+	
3-Methyl-2-butanol (Alc)	+		
1,3,5-Trioxane (**–**)	+		
3-Pentanone (K)	+		
2,5-Dimethylfuran (F)	+		
2,4,4-Trimethyl-1-pentene (H)	+		
2-Methyl-3-pentanone (K)	+		
3-Ethyl-2-methylpentane (H)	+		
Cycloheptatriene (H)	+	+	
2-Methyl-3-pentanol (Alc)	+		
Octane (H)	+	+	
2,3-Dimethylbutanoic acid methyl ester (Es)	+		
3-Methylhexanal (Ald)	+		
Ethylbenzene (H)	+		
Xylene (H)	+		
Nonane (H)	+	+	R [161]
Methyl furan-3-carboxylate (F)	+		
3-Ethyl-2-methyl-3-pentanol (Alc)	+		
Ethyltoluene (H)	+		
Methyl 2-furoate (Es)	+	+	
3-Octanone (K)	+		SR [162]
2-Pentylfuran (F)	+		R [163]
(1,2-Dimethylpropyl)cyclopropane (**–**)	+		
Phthalic anhydride	+		
Selinene (T)	+		
Cedrene (T)	+		
Longipinene (T)	+		
Thujopsene (T)	+		
Cuparene (T)	+		
Cadinene (T)	+	+	
Diphenylphenol (Alc)	+		

Compound class: (Alc) Alcohols; (Ald) Aldehydes; (Es) Esters; (F) Furans; (H) Hydrocarbons; (K) Ketones; (T) Terpenes; (**–**) undescribed. Interaction: (A) insect attraction; (R) insect repellent; (SR) terrestrial molluscs repellent

**Table 5:** Volatile compounds of *P. ostreatus* when grown on sugarcane bagasse [78], wheat straw [164] or Raper medium [78] in the absence of mushroom formation

Compound (Class)	Ligno-cellulose	Raper medium	Interaction (Reference)
1-Heptene (H)	+		
2-Methylbutanol (Alc)	+		
1-Hexanal (Ald)	+		
1-Octene (H)	+		SR [162]
1,3-Octadiene (H)	+		
α-Pinene (T)	+		A [165]; R [166]
2-Octen-3-one (K)	+	+	
1-Octen-3-ol (Alc)	+	+	A [80, 81]; A & R [82]; SR [162]
3-Octanone (K)	+	+	SR [162]
3-Octanol (Alc)	+	+	
Octanal (Ald)	+		A [167]
2-Octenol (Alc)	+	+	
1-Octanol (Alc)	+	+	
4-Methoxybenzaldehyde (Ald)	+		R [168]

Compound classes: (Alc) Alcohols; (Ald) Aldehydes; (H) Hydrocarbons; (K) Ketones; (T) Terpenes. Insect interaction: (A) attraction; (R) insect repellent; (SR) terrestrial molluscs repellent

## Recommendations

Fungal materials have attracted a lot of interest during the last decade, showing analogy with the interest in collected and cultivated edible mushrooms. The latter prompted the Nordic countries to re-assess safety of mushrooms that are being traded and / or collected and consumed by individuals [90]. Clearly, risk assessment of fungi for materials is different from that of consumption but we can learn from such assessments.

The list of species used or proposed to make mycelium materials (Table 1) is a fraction of the (wood degrading) fungi found in nature. Effective screens will be needed to unravel the potential of fungal species to produce mycelium materials. So far, only three publications [11, 91, 92] reported screening of ≥ 10 species for their applicability to make composite materials. This should be scaled up selecting not only for mechanical properties but also for instance for rate of colonization, robustness of performance, the amount of CO_2_ emission, visual appearance and haptic properties, genetic stability of the strain, and the ability to easily maintain stocks of the strains. Several methods have still to be developed, while others have been established such as those to quantify mechanical properties [92], to store fungi [93], or to monitor homogeneity and rate of colonization in a 3D substrate [94].

On top of the mechanical and other performative properties, fungi should be screened based on a risk assessment. Such a risk assessment could be partly based on a history of safe use, for instance as a food. Risk assessment relies on a correct identification of the fungal species that is used. Therefore, standards of identification should be used such as proposed recently [95]. This identification should be linked to biosafety. In particular, fungi should be selected that can be used at Bio Safety Level 1 during production of the mycelium materials (see [96, 97]). Pathogenic fungi and fungi producing mycotoxins should not be used when the final product contains living mycelium. In addition, species should be avoided that attract insects when grown in certain substrates. Still, little is known about release of volatiles by fungi and their impact on insect communities. Species that are commonly used to produce fungal materials have been selected based on their speed of colonizing substrates, thereby being potentially highly competitive in nature. Especially when exotic fungi are used they may become invasive, replacing part of the local fungal communities [98].

Fruiting should be repressed during formation and later use of mycelium materials to prevent spreading in the natural environment. Mushroom production in *Coprinopsis cinerea* and *Pleurotus djamor* is repressed by glycogen synthase kinase-3 (GSK-3) inhibitors like lithium chloride and CHIR99021 trihydrochloride. These inhibitors have been proposed to be included in the substrates to inhibit fruiting body formation on fungal materials and regulating mycelium growth [99]. Alternatively, one could use non-sporulating strains. Such strains are already being used in the cultivation of *P. ostreatus* to protect workers from exposure to spores [36, 55]. Clearly, the most practical solution to prevent spreading in the natural environment is to kill the fungus before it leaves the production facility. Use of local strains not only prevents introduction of invasive species, it also is the easiest way to comply with the Nagoya protocol. This protocol was put in place in 2014 to enforce fair and equitable sharing of benefits arising from the use of genetic resources from countries [100].

When working with fungi standardisation is key [9]. This is not only essential to compare screens in different laboratories but also to ensure reproducible manufacturing and material properties. For instance, drying of mycelium materials should be standardized. So far, drying is done at room temperature, in an oven, or a drier. An important aspect is the insulation property of mycelium composites, with surrounding material potentially keeping the inner section viable and/or moist. Therefore, studies should unveil viability of mycelium composites after drying and heat treatment by plating and counting colony-forming units. Possibly, substrates, species and materials dimensions as well as methods of drying should be optimized. In addition, studies have to be performed to demonstrate stability of the material in time. Only recently an article assessed the impact of tropical weathering conditions (75 ± 15% relative humidity and 27.5 ± 2.5 °C) on the mechanical properties of composite material [101]. Mechanical properties of uncoated samples substantially dropped over 35 days, whereas applying an oil-based coating reduced the weathering effect, albeit only significant for tensile strength. This was explained by the high porosity of the composite material that prevented the coating from forming a perfect sealed surface, thus enabling moisture from entering. Finally, biodegradability of the mycelium material after use should be assessed as well as the bioavailability of the nutrients contained in the material. So far, these topics have not been addressed in the literature.

Together, the following recommendations are made.

- Fungi used for fungal materials should be identified via standardized procedures;
- Use standardized methods to select fungal species and strains for specific applications;
- Fungi should be selected that are not pathogenic to humans, animals and plants;
- Fungi should be selected that do not produce mycotoxins, even when the fungal materials have non-food applications;
- Use spore-less strains;
- Preferably use local fungi to produce mycelium materials;
- Work at biological safety levels as dictated by the local authorities;
- Preferably kill the fungus in the mycelium material before it is leaves the production facility;
- Regularly confirm efficacy of the killing procedure.

## Conclusions

**Fig. 1:**
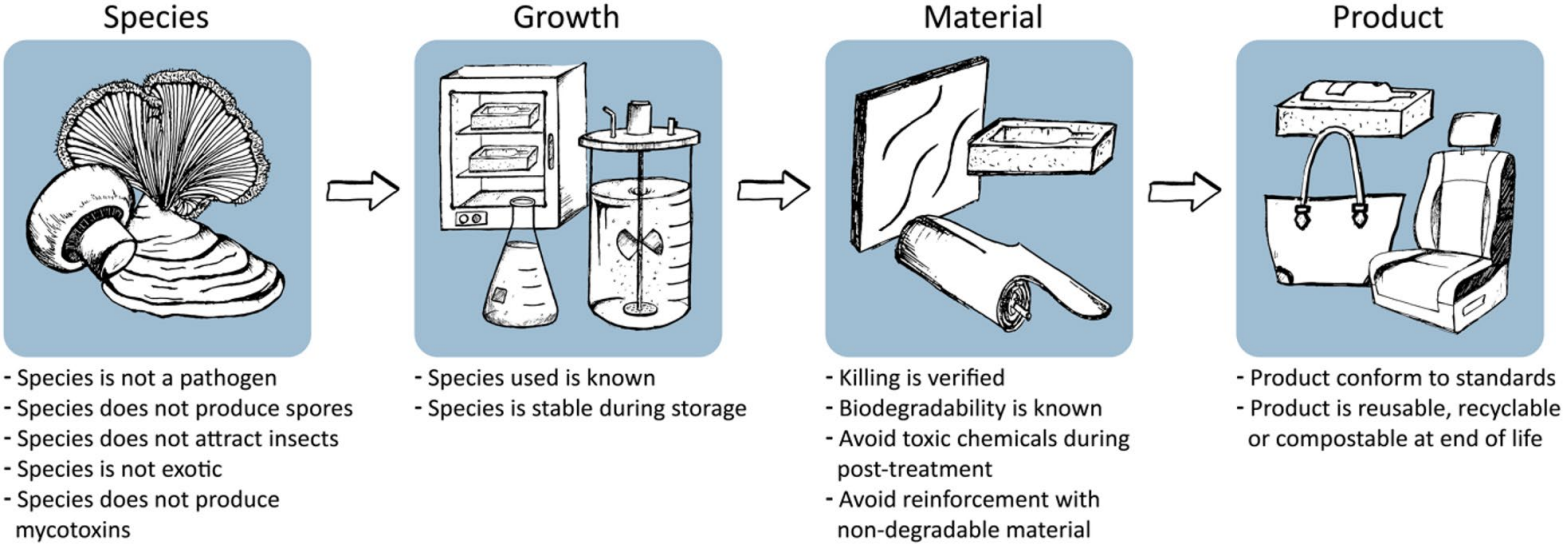
Risk mitigation of fungal material processing

Fungal materials have a very high potential to replace non-sustainable products on the market. In fact, fungal materials may even have properties that are not yet provided by other materials. Given their potential, fungal materials may be used at a very large scale. In the future, people may be surrounded by these materials in their houses, at work, and may even wear it. This requires a critical assessment of the risks associated with fungal materials. This includes the selection of species used for making the materials, the conditions used during the production process and when they leave the production facility, as well as measures to prevent impact on the environment when the products are used in society (Fig. 1). Our assessment of pathogenicity and mycotoxin data indicates that fungal species that have been described in scientific publications to produce fungal materials show low risk, if at all, for workers, consumers and the environment.

Genetic modification could be used to improve properties of mycelium materials, and to reduce pathogenicity, invasiveness, mycotoxin production, spreading in the environment, and/or attraction by insects. In this case too, introduction on the market should be accompanied by a risk assessment. This would be particularly needed when the mycelium product contains a living fungus.

## Data Availability

Not applicable.

## References

[1] United Nations. https://www.un.org/development/desa/en/news/population/world-population-prospects-2019.html.

[2] Stahel WR (2016). The circular economy. Nature.

[3] Plastics Europe. Plastics–The Facts 2021 an Analysis of European Plastics Production, Demand and Waste Data; Plastics Europe: Brussels, Belgium, 2021. https://plasticseurope.org/knowledge-hub/plastics-the-facts-2021/

[4] Geyer R, Jambeck JR, Law KL (2017). Production, use, and fate of all plastics ever made. Sci Adv.

[5] Sandin G, Peters GM (2018). Environmental impact of textile reuse and recycling—a review. J Clean Prod.

[6] China CR, Maguta MM, Nyandoro SS, Hilonga A, Kanth SV, Njau KN (2020). Alternative tanning technologies and their suitability in curbing environmental pollution from the leather industry: a comprehensive review. Chemosphere.

[7] IEA. Cement, IEA, Paris. 2021. https://www.iea.org/reports/cement. Accessed 15 Nov 2021.

[8] Grimm D, Wösten HAB (2018). Mushroom cultivation in the circular economy. Appl Microbiol Biotechnol.

[9] Meyer V, Basenko EY, Benz JP, Braus GH, Caddick MX, Csukai M (2020). Growing a circular economy with fungal biotechnology: a white paper. Fungal Biol Biotechnol.

[10] Forbes. Fungi fashion is booming as Adidas launches new mushroom leather shoe. 2021. https://www.forbes.com/sites/annahaines/2021/04/15/fungi-fashion-is-booming-as-adidas-launches-new-mushroom-leather-shoe/. Accessed 22 Oct 2021.

[11] Jones M, Huynh T, John S (2018). Inherent species characteristic influence and growth performance assessment for mycelium composite applications. Adv Mater Lett.

[12] Appels FVW, Camere S, Montalti M, Karana E, Jansen KMB, Dijksterhuis J (2019). Fabrication factors influencing mechanical, moisture- and water-related properties of mycelium-based composites. Mater Des.

[13] Jones M, Mautner A, Luenco S, Bismarck A, John S (2020). Engineered mycelium composite construction materials from fungal biorefineries: a critical review. Mater Des.

[14] Santos IS, Nascimento BL, Marino RH, Sussuchi EM, Matos MP, Griza S (2021). Influence of drying heat treatments on the mechanical behavior and physico-chemical properties of mycelial biocomposite. Compos B Eng.

[15] McBee RM, Lucht M, Mukhitov N, Richardson M, Srinivasan T, Meng D (2021). Engineering living and regenerative fungal–bacterial biocomposite structures. Nat Mater.

[16] Pelletier MG, Holt GA, Wanjura JD, Bayer E, McIntyre G (2013). An evaluation study of mycelium based acoustic absorbers grown on agricultural by-product substrates. Ind Crops Prod.

[17] Adamatzky A, Gandia A (2021). Living mycelium composites discern weights via patterns of electrical activity. J Biores Bioprod.

[18] Elsacker E, Søndergaard A, Van Wylick A, Peeters E, De Laet L (2021). Growing living and multifunctional mycelium composites for large-scale formwork applications using robotic abrasive wire-cutting. Constr Build Mater.

[19] Modanloo B, Ghazvinian A, Matini M, Andaroodi E (2021). Tilted arch; implementation of additive manufacturing and bio-welding of mycelium-based composites. Biomimetics.

[20] Pelletier MG, Holt GA, Wanjura JD, Greetham L, McIntyre G, Bayer E (2019). Acoustic evaluation of mycological biopolymer, an all-natural closed cell foam alternative. Ind Crops Prod.

[21] Nawawi WM, Lee KY, Kontturi E, Murphy RJ, Bismarck A (2019). Chitin nanopaper from mushroom extract: natural composite of nanofibers and glucan from a single biobased source. ACS Sustain Chem Eng.

[22] Nawawi WM, Lee KY, Kontturi E, Bismarck A, Mautner A (2020). Surface properties of chitin-glucan nanopapers from *Agaricus bisporus*. Int J Biol Macromol.

[23] Nawawi WM, Jones MP, Kontturi E, Mautner A, Bismarck A (2020). Plastic to elastic: fungi-derived composite nanopapers with tunable tensile properties. Compos Sci Technol.

[24] Gandia A, van den Brandhof JG, Appels FVW, Jones MP (2021). Flexible fungal materials: shaping the future. Trends Biotechnol.

[25] Appels FVW, Dijksterhuis J, Lukasiewicz CE, Jansen KMB, Wösten HAB, Krijgsheld P (2018). Hydrophobin gene deletion and environmental growth conditions impact mechanical properties of mycelium by affecting the density of the material. Sci Rep.

[26] César E, Canche-Escamilla G, Montoya L, Ramos A, Duarte-Aranda S, Bandala VM (2021). Characterization and physical properties of mycelium films obtained from wild fungi: natural materials for potential biotechnological applications. J Polym Environ.

[27] Appels FVW, van den Brandhof JG, Dijksterhuis J, de Kort GW, Wösten HAB (2020). Fungal mycelium classified in different material families based on glycerol treatment. Commun Biol.

[28] Adamatzky A, Tegelaar M, Wosten HAB, Powell AL, Beasley AE, Mayne R (2020). On Boolean gates in fungal colony. Biosystems.

[29] Adamatzky A, Gandia A, Chiolerio A (2021). Towards fungal sensing skin. Fungal Biol Biotechnol.

[30] Naranjo-Ortiz MA, Gabaldón T (2019). Fungal evolution: diversity, taxonomy and phylogeny of the fungi. Biol Rev.

[31] Bánki O, Roskov Y, Vandepitte L, DeWalt RE, Remsen D, Schalk P, et al. Catalogue of life checklist (Version 2021-09-21). 2021. 10.48580/d4sv

[32] Moore D, Robson GD, Trinci AP. 21st century guidebook to fungi. Cambridge University Press. 2011. ISBN: 978-1-107-00676-8

[33] Wu B, Hussain M, Zhang W, Stadler M, Liu X, Xiang M (2019). Current insights into fungal species diversity and perspective on naming the environmental DNA sequences of fungi. Mycology.

[34] Baumgartner K, Coetzee MP, Hoffmeister D (2011). Secrets of the subterranean pathosystem of Armillaria. Mol Plant Pathol.

[35] Brown GD, Denning DW, Levitz SM (2012). Tackling human fungal infections. Science.

[36] Baars JJP, Hendrickx PM, Sonnenberg ASM (2004). Prototype of a sporeless oyster mushroom. Mushroom Sci.

[37] Munoz P, Bouza E, Cuenca-Estrella M, Eiros JM, Pérez MJ, Sánchez-Somolinos M (2005). *Saccharomyces cerevisiae* fungemia: an emerging infectious disease. Clin Infect Dis.

[38] Ohm RA, Riley R, Salamov A, Min B, Choi IG, Grigoriev IV (2014). Genomics of wood-degrading fungi. Fungal Genet Biol.

[39] Fukasawa Y (2021). Ecological impacts of fungal wood decay types: a review of current knowledge and future research directions. Ecol Res.

[40] Sieber TN (2007). Endophytic fungi in forest trees: are they mutualists?. Fungal Biol Rev.

[41] Saikkonen K (2007). Forest structure and fungal endophytes. Fungal Biol Rev.

[42] Xia Y, Sahib MR, Amna A, Opiyo SO, Zhao Z, Gao YG (2019). Culturable endophytic fungal communities associated with plants in organic and conventional farming systems and their effects on plant growth. Sci Rep.

[43] Fröhlich-Nowoisky J, Pickersgill DA, Després VR, Pöschl U (2009). High diversity of fungi in air particulate matter. Proc Natl Acad Sci.

[44] Baum S, Sieber TN, Schwarze FW, Fink S (2003). Latent infections of *Fomes fomentarius* in the xylem of European beech (*Fagus sylvatica*). Mycol Prog.

[45] Paterson RRM (2007). *Ganoderma* disease of oil palm—a white rot perspective necessary for integrated control. Crop Prot.

[46] van den Brule T, Punt M, Teertstra W, Houbraken J, Wösten HAB, Dijksterhuis J (2020). The most heat-resistant conidia observed to date are formed by distinct strains of *Paecilomyces variotii*. Environ Microbiol.

[47] Tournas V (1994). Heat-resistant fungi of importance to the food and beverage industry. Crit Rev Microbiol.

[48] Cortesão M, de Haas A, Unterbusch R, Fujimori A, Schütze T, Meyer V, Moeller R (2020). *Aspergillus niger* spores are highly resistant to space radiation. Front Microbiol.

[49] Ijadpanahsaravi M, Punt M, Wösten HAB, Teertstra WR (2021). Minimal nutrient requirements for induction of germination of *Aspergillus **niger* conidia. Fungal Biol.

[50] Wyatt TT, Wösten HAB, Dijksterhuis J (2013). Fungal spores for dispersion in space and time. Adv Appl Microbiol.

[51] Xie Y, Chang J, Kwan HS (2020). Carbon metabolism and transcriptome in developmental paths differentiation of a homokaryotic *Coprinopsis cinerea* strain. Fungal Genet Biol.

[52] Fisher MC, Henk DA, Briggs CJ, Brownstein JS, Madoff LC, McCraw SL, Gurr SJ (2012). Emerging fungal threats to animal, plant and ecosystem health. Nature.

[53] Dressaire E, Yamada L, Song B, Roper M (2016). Mushrooms use convectively created airflows to disperse their spores. Proc Natl Acad Sci.

[54] Drenth A, Kema GHJ (2021). The vulnerability of bananas to globally emerging disease threats. Phytopathology.

[55] Lavrijssen B, Baars JP, Lugones LG, Scholtmeijer K, Sedaghat Telgerd N, Sonnenberg AS (2020). Interruption of an *MSH4* homolog blocks meiosis in metaphase I and eliminates spore formation in *Pleurotus ostreatus*. PLoS ONE.

[56] Chowdhary A, Randhawa HS, Gaur SN, Agarwal K, Kathuria S, Roy P (2012). *Schizophyllum commune* as an emerging fungal pathogen: a review and report of two cases. Mycoses.

[57] Kainz K, Bauer MA, Madeo F, Carmona-Gutierrez D (2020). Fungal infections in humans: the silent crisis. Microb Cell.

[58] Dean R, Van Kan JA, Pretorius ZA, Hammond-Kosack KE, Di Pietro A, Spanu PD (2012). The Top 10 fungal pathogens in molecular plant pathology. Mol Plant Pathol.

[59] Ryvarden, L, Melo I. Poroid fungi of Europe 2nd ed. Oslo: Fungiflora; 2017. ISBN: 978-82-90724-54-7

[60] Bernicchia A, Gorjón SP. Polypores of the Mediterranean region. Romar; 2020. ISBN: 978-88-96182-14-7

[61] Susanto A, Sudharto PS, Purba RY (2005). Enhancing biological control of basal stem rot disease (*Ganoderma boninense*) in oil palm plantations. Mycopathologia.

[62] Sankaran KV, Bridge PD, Gokulapalan C (2005). *Ganoderma* diseases of perennial crops in India–an overview. Mycopathologia.

[63] Vinjusha N, Arun Kumar TK (2021). Revision of *Ganoderma* species associated with stem rot of coconut palm. Mycologia.

[64] Fischer M (2006). Biodiversity and geographic distribution of basidiomycetes causing esca-associates white rot in grapevine: a worldwide perspective. Phytopathol Mediterr.

[65] Fischer M, González GV (2015). An annotated checklist of European basidiomycetes related to white rot of grapevine (*Vitis vinifera*). Phytopathol Mediterr.

[66] Adaskaveg JE, Ogawa JM (1990). Wood decay pathology of fruit and nut trees in California. Plant Dis.

[67] Gabriel J, Švec K (2017). Occurrence of indoor wood decay basidiomycetes in Europe. Fungal Biol Rev.

[68] Farr DF, Rossman AY. Fungal Databases, U.S. National Fungus Collections, ARS, USDA. https://nt.ars-grin.gov/fungaldatabases/. Accessed 8 Nov 2021.

[69] Baranova MA, Logacheva MD, Penin AA, Seplyarskiy VB, Safonova YY, Naumenko SA (2015). Extraordinary genetic diversity in a wood decay mushroom. Mol Biol Evol.

[70] Paterson RRM (2006). Fungi and fungal toxins as weapons. Mycol Res.

[71] Klassen-Fischer MK (2006). Fungi as bioweapons. Clin Lab Med.

[72] de Mattos-Shipley KM, Ford KL, Alberti F, Banks AM, Bailey AM, Foster GD (2016). The good, the bad and the tasty: the many roles of mushrooms. Stud Mycol.

[73] Riley R, Salamov AA, Brown DW, Nagy LG, Floudas D, Held BW (2014). Extensive sampling of basidiomycete genomes demonstrates inadequacy of the white-rot/brown-rot paradigm for wood decay fungi. Proc Natl Acad Sci.

[74] Enjalbert F, Cassanas G, Rapior S, Renault C, Chaumont JP (2004). Amatoxins in wood-rotting *Galerina marginata*. Mycologia.

[75] Boonen J, Malysheva SV, Taevernier L, Diana Di Mavungu J, De Saeger S, De Spiegeleer B (2012). Human skin penetration of selected model mycotoxins. Toxicology.

[76] Biedermann PH, Vega FE (2020). Ecology and evolution of insect–fungus mutualisms. Annu Rev Entomol.

[77] Korpi A, Järnberg J, Pasanen AL (2009). Microbial volatile organic compounds. Crit Rev Toxicol.

[78] Kabbaj W, Breheret S, Guimberteau J, Talou T, Olivier JM, Bensoussan M (2002). Comparison of volatile compound production in fruit body and in mycelium of *Pleurotus ostreatus* identified by submerged and solid-state cultures. Appl Biochem Biotechnol.

[79] Štefániková J, Martišová P, Šnirc M, Kunca V, Árvay J (2021). The effect of *Amanita rubescens* Pers developmental stages on aroma profile. J Fungi.

[80] Morath SU, Hung R, Bennett JW (2012). Fungal volatile organic compounds: a review with emphasis on their biotechnological potential. Fungal Biol Rev.

[81] Inamdar AA, Morath S, Bennett JW (2020). Fungal volatile organic compounds: more than just a funky smell?. Annu Rev Microbiol.

[82] Davis TS, Crippen TL, Hofstetter RW, Tomberlin JK (2013). Microbial volatile emissions as insect semiochemicals. J Chem Ecol.

[83] Schiestl FP, Steinebrunner F, Schulz C, Von Reuss S, Francke W, Weymuth C (2006). Evolution of ‘pollinator’-attracting signals in fungi. Biol Lett.

[84] Fäldt J, Jonsell M, Nordlander G, Borg-Karlson AK (1999). Volatiles of bracket fungi *Fomitopsis pinicola* and *Fomes fomentarius* and their functions as insect attractants. J Chem Ecol.

[85] Witte V, Maschwitz U (2008). Mushroom harvesting ants in the tropical rain forest. Naturwissenschaften.

[86] von Beeren C, Mair MM, Witte V (2014). Discovery of a second mushroom harvesting ant (Hymenoptera: Formicidae) in Malayan tropical rainforests. Myrmecol News.

[87] Epps MJ, Penick CA (2018). Facultative mushroom feeding by common woodland ants (Formicidae, *Aphaenogaster* spp.). Food Webs.

[88] Bruce TJ, Pickett JA (2011). Perception of plant volatile blends by herbivorous insects–finding the right mix. Phytochemistry.

[89] Bajwa DS, Holt GA, Bajwa SG, Duke SE, McIntyre G (2017). Enhancement of termite (*Reticulitermes flavipes* L.) resistance in mycelium reinforced biofiber-composites. Ind Crops Prod.

[90] Gry J, Andersson C, Krüger L, Lyrån B (2012). Mushrooms traded as food.

[91] Wimmers G, Klick J, Tackaberry L, Zwiesigk C, Egger K, Massicotte H (2019). Fundamental studies for designing insulation panels from wood shavings and filamentous fungi. BioResources.

[92] Appels FVW. The use of fungal mycelium for the production of bio-based materials. 2020. ISBN: 978-94-6380-683-1. https://dspace.library.uu.nl/handle/1874/390884

[93] Nakasone KK, Peterson SW, Jong SC. Preservation and distribution of fungal cultures. Biodiversity of fungi: inventory and monitoring methods. Amsterdam: Elsevier Academic Press. 2004;3:37–47.

[94] Herman KC, Wösten HAB, Fricker MD, Bleichrodt RJ (2020). Growth induced translocation effectively directs an amino acid analogue to developing zones in *Agaricus bisporus*. Fungal Biol.

[95] Lücking R, Aime MC, Robbertse B, Miller AN, Ariyawansa HA, Aoki T (2020). Unambiguous identification of fungi: where do we stand and how accurate and precise is fungal DNA barcoding?. IMA Fungus.

[96] World Federation of Culture Collections. www.wfcc.info

[97] American Type Culture Collection. www.attc.org

[98] Nordén J, Abrego N, Boddy L, Bässler C, Dahlberg A, Halme P (2020). Ten principles for conservation translocations of threatened wood-inhabiting fungi. Fungal Ecol.

[99] Chang J, Chan PL, Xie Y, Ma KL, Cheung MK, Kwan HS (2019). Modified recipe to inhibit fruiting body formation for living fungal biomaterial manufacture. PLoS ONE.

[100] Overmann J, Scholz AH (2017). Microbiological research under the Nagoya Protocol: facts and fiction. Trends Microbiol.

[101] Chan XY, Saeidi N, Javadian A, Hebel DE, Gupta M (2021). Mechanical properties of dense mycelium-bound composites under accelerated tropical weathering conditions. Sci Rep.

[102] Větrovský T, Morais D, Kohout P, Lepinay C, Algora C, Hollá SA (2020). GlobalFungi, a global database of fungal occurrences from high-throughput-sequencing metabarcoding studies. Sci Data.

[103] Dai YC, Cui BK, Yuan HS, Li BD (2007). Pathogenic wood-decaying fungi in China. For Pathol.

[104] Cartabia M, Girometta CE, Milanese C, Baiguera RM, Buratti S, Branciforti DS (2021). Collection and characterization of wood decay fungal strains for developing pure mycelium mats. J Fungi.

[105] Tacer-Caba Z, Varis JJ, Lankinen P, Mikkonen KS (2020). Comparison of novel fungal mycelia strains and sustainable growth substrates to produce humidity-resistant biocomposites. Mater Des.

[106] Janesch J, Jones M, Bacher M, Kontturi E, Bismarck A, Mautner A (2020). Mushroom-derived chitosan-glucan nanopaper filters for the treatment of water. React Funct Polym.

[107] Jones M, Weiland K, Kujundzic M, Theiner J, Kählig H, Kontturi E (2019). Waste-derived low-cost mycelium nanopapers with tunable mechanical and surface properties. Biomacromol.

[108] Ifuku S, Nomura R, Morimoto M, Saimoto H (2011). Preparation of chitin nanofibers from mushrooms. Materials.

[109] Hamlyn PF, Schmidt RJ (1994). Potential therapeutic application of fungal filaments in wound management. Mycologist.

[110] Attias N, Danai O, Ezov N, Tarazi E, Grobman YJ. Developing novel applications of mycelium based bio-composite materials for design and architecture. Proceedings of Building with Biobased Materials: Best practice and Performance Specification, 2017;76–7.

[111] Shao GB, Yang P, Jiang WX. Research and preparation of mycelium-soybean straw composite materials. 2nd Annual International Conference on Advanced Material Engineering. 2016:9–15. 10.2991/ame-16.2016.2

[112] Papp N, Rudolf K, Bencsik T, Czégényi D (2017). Ethnomycological use of *Fomes fomentarius* (L.) Fr and *Piptoporus betulinus* (Bull.) P. Karst. in Transylvania, Romania. Genet Resour Crop Evol.

[113] Pegler DN (2001). Useful fungi of the world: Amadou and Chaga. Mycologist.

[114] Müller C, Klemm S, Fleck C (2021). Bracket fungi, natural lightweight construction materials: hierarchical microstructure and compressive behavior of *Fomes fomentarius* fruit bodies. Appl Phys A.

[115] Stelzer L, Hoberg F, Bach V, Schmidt B, Pfeiffer S, Meyer V (2021). Life cycle assessment of fungal-based composite bricks. Sustainability.

[116] Vallas T, Courard L (2017). Using nature in architecture: building a living house with mycelium and trees. Front Archit Res.

[117] Soh E, Chew ZY, Saeidi N, Javadian A, Hebel D, Le Ferrand H (2020). Development of an extrudable paste to build mycelium-bound composites. Mater Des.

[118] Silverman J, Cao H, Cobb K (2020). Development of mushroom mycelium composites for footwear products. Cloth Text Res J.

[119] Liu R, Long L, Sheng Y, Xu J, Qiu H, Li X, Wang Y, Wu H (2019). Preparation of a kind of novel sustainable mycelium/cotton stalk composites and effects of pressing temperature on the properties. Ind Crops Prod.

[120] Liu R, Li X, Long L, Sheng Y, Xu J, Wang Y (2020). Improvement of mechanical properties of mycelium/cotton stalk composites by water immersion. Compos Interfaces.

[121] Răut I, Călin M, Vuluga Z, Oancea F, Paceagiu J, Radu N (2021). Fungal based biopolymer composites for construction materials. Materials.

[122] Rigobello A, Ayres P (2021). Mycelium-based composites as two-phase particulate composites: compressive behaviour of anisotropic designs. Res Square..

[123] Soh E, Saeidi N, Javadian A, Hebel DE, Le Ferrand H (2021). Effect of common foods as supplements for the mycelium growth of *Ganoderma lucidum* and *Pleurotus ostreatus* on solid substrates. PLoS ONE.

[124] Antinori ME, Ceseracciu L, Mancini G, Heredia-Guerrero JA, Athanassiou A (2020). Fine-tuning of physicochemical properties and growth dynamics of mycelium-based materials. ACS Appl Bio Mater.

[125] Haneef M, Ceseracciu L, Canale C, Bayer IS, Heredia-Guerrero JA, Athanassiou A (2017). Advanced materials from fungal mycelium: fabrication and tuning of physical properties. Sci Rep.

[126] Antinori ME, Contardi M, Suarato G, Armirotti A, Bertorelli R, Mancini G (2021). Advanced mycelium materials as potential self-growing biomedical scaffolds. Sci Rep.

[127] Elsacker E, Vandelook S, Brancart J, Peeters E, De Laet L (2019). Mechanical, physical and chemical characterisation of mycelium-based composites with different types of lignocellulosic substrates. PLoS ONE.

[128] Angelova G, Brazkova M, Stefanova P, Blazheva D, Vladev V, Petkova N (2021). Waste rose flower and lavender straw biomass—an innovative lignocellulose feedstock for mycelium bio-materials development using newly isolated *Ganoderma resinaceum* GA1M. J Fungi.

[129] Attias N, Danai O, Abitbol T, Tarazi E, Ezov N, Pereman I, Grobman YJ (2019). Mycelium bio-composites in industrial design and architecture: comparative review and experimental analysis. J Clean Prod.

[130] Yang Z, Zhang F, Still B, White M, Amstislavski P (2017). Physical and mechanical properties of fungal mycelium-based biofoam. J Mater Civ Eng.

[131] Bruscato C, Malvessi E, Brandalise RN, Camassola M (2019). High performance of macrofungi in the production of mycelium-based biofoams using sawdust—sustainable technology for waste reduction. J Clean Prod.

[132] Bustillos J, Loganathan A, Agrawal R, Gonzalez BA, Perez MG, Ramaswamy S (2020). Uncovering the mechanical, thermal, and chemical characteristics of biodegradable mushroom leather with intrinsic antifungal and antibacterial properties. ACS Appl Bio Mater.

[133] Teixeira JL, Matos MP, Nascimento BL, Griza S, Holanda FS, Marino RH (2018). Production and mechanical evaluation of biodegradable composites by white rot fungi. Cienc e Agrotecnologia.

[134] Sivaprasad S, Byju SK, Prajith C, Shaju J, Rejeesh CR (2021). Development of a novel mycelium bio-composite material to substitute for polystyrene in packaging applications. Mater Today: Proc.

[135] Nashiruddin NI, Chua KS, Mansor AF, Rahman AR, Lai JC, Wan Azelee NI, El Enshasy H (2021). Effect of growth factors on the production of mycelium-based biofoam. Clean Technol Environ Policy.

[136] Zhang J, He P, Lin Y, Song H, Dong H, Zhu X, Zhang J (2019). The cushion performance of mycelium-cornstraw biofoams. J Biobased Mater Bioenergy.

[137] He J, Cheng CM, Su DG, Zhong MF (2014). Study on the mechanical properties of the latex-mycelium composite. Appl Mech Mater.

[138] Helberg J, Klöcker M, Sabantina L, Storck JL, Böttjer R, Brockhagen B (2019). Growth of *Pleurotus ostreatus* on different textile materials for vertical farming. Materials.

[139] Kuribayashi T, Lankinen P, Hietala S, Mikkonen KS (2022). Dense and continuous networks of aerial hyphae improve flexibility and shape retention of mycelium composite in the wet state. Compos A: Appl Sci Manuf.

[140] Trabelsi M, Mamun A, Klöcker M, Brockhagen B, Kinzel F, Kapanadze D, Sabantina L (2021). Polyacrylonitrile (PAN) nanofiber mats for mushroom mycelium growth investigations and formation of mycelium-reinforced nanocomposites. J Eng Fibers Fabr.

[141] Jones MP, Lawrie AC, Huynh TT, Morrison PD, Mautner A, Bismarck A, John S (2019). Agricultural by-product suitability for the production of chitinous composites and nanofibers utilising *Trametes versicolor* and *Polyporus brumalis* mycelial growth. Process Biochem.

[142] Wijayarathna EK, Mohammadkhani G, Soufiani AM, Adolfsson KH, Ferreira JA, Hakkarainen M (2021). Fungal textile alternatives from bread waste with leather-like properties. Resour Conserv Recycl.

[143] Lugones LG, De Jong JF, De Vries OM, Jalving R, Dijksterhuis J, Wösten HAB (2004). The SC15 protein of *Schizophyllum commune* mediates formation of aerial hyphae and attachment in the absence of the SC3 hydrophobin. Mol Microbiol.

[144] Attias N, Reid M, Mijowska SC, Dobryden I, Isaksson M, Pokroy B (2021). Biofabrication of nanocellulose–mycelium hybrid materials. Adv Sustain Syst.

[145] Jones M, Bhat T, Huynh T, Kandare E, Yuen R, Wang CH, John S (2018). Waste-derived low-cost mycelium composite construction materials with improved fire safety. Fire Mater.

[146] Jones M, Bhat T, Kandare E, Thomas A, Joseph P, Dekiwadia C, Yuen R, John S, Ma J, Wang CH (2018). Thermal degradation and fire properties of fungal mycelium and mycelium-biomass composite materials. Sci Rep.

[147] Koc B, Akyuz L, Cakmak YS, Sargin I, Salaberria AM, Labidi J (2020). Production and characterization of chitosan-fungal extract films. Food Biosci.

[148] NDFF. NDFF Verspreidingsatlas. www.verspreidingsatlas.nl. Accessed 5 Nov 2021.

[149] van der Vlugt RA, van Raaij H, de Weerdt M, Bergervoet JH (2015). Multiplex detection of plant pathogens through the Luminex MagPlex bead system. Methods Mol Biol.

[150] Boddy L, Heilmann-Clausen J (2008). Basidiomycete community development in temperate angiosperm wood. Brit Mycol Soc Symposia Series.

[151] Schwartze VU, Winter S, Shelest E, Marcet-Houben M, Horn F, Wehner S (2014). Gene expansion shapes genome architecture in the human pathogen *Lichtheimia corymbifera*: an evolutionary genomics analysis in the ancient terrestrial mucorales (Mucoromycotina). PLoS Genet.

[152] Robert V, Stegehuis G, Stalpers, J. The MycoBank engine and related databases. 2005. https://www.mycobank.org/. Accessed 5 Nov 2021.

[153] Jouda JB, Njoya EM, Mbazoa CD, Zhou Z, Lannang AM, Wandji J (2018). Lambertellin from *Pycnoporus sanguineus* MUCL 51321 and its anti-inflammatory effect via modulation of MAPK and NF-κB signaling pathways. Bioorg Chem.

[154] Lukács G, Papp T, Nyilasi I, Nagy E, Vágvölgyi C (2004). Differentiation of *Rhizomucor* species on the basis of their different sensitivities to lovastatin. J Clin Microbiol.

[155] Liu M, Bruni GO, Taylor CM, Zhang Z, Wang P (2018). Comparative genome-wide analysis of extracellular small RNAs from the mucormycosis pathogen *Rhizopus delemar*. Sci Rep.

[156] Takemoto S, Nakamura H, Imamura Y, Shimane T (2010). *Schizophyllum commune* as a ubiquitous plant parasite. Jpn Agric Res Q.

[157] Rashmi M, Kushveer JS, Sarma VV (2019). A worldwide list of endophytic fungi with notes on ecology and diversity. Mycosphere.

[158] Oses R, Valenzuela S, Freer J, Sanfuentes E, Rodriguez J (2008). Fungal endophytes in xylem of healthy Chilean trees and their possible role in early wood decay. Fungal Divers.

[159] Konuma R, Umezawa K, Mizukoshi A, Kawarada K, Yoshida M (2015). Analysis of microbial volatile organic compounds produced by wood-decay fungi. Biotechnol Lett.

[160] Schalchli H, Hormazábal E, Astudillo Á, Briceño G, Rubilar O, Diez MC (2021). Bioconversion of potato solid waste into antifungals and biopigments using *Streptomyces* spp.. PLoS ONE.

[161] Schoelitsz B, Mwingira V, Mboera LE, Beijleveld H, Koenraadt CJ, Spitzen J (2020). Chemical mediation of oviposition by *Anopheles* mosquitoes: a push-pull system driven by volatiles associated with larval stages. J Chem Ecol.

[162] Khoja S, Eltayef KM, Baxter I, Bull JC, Loveridge EJ, Butt T (2019). Fungal volatile organic compounds show promise as potent molluscicides. Pest Manag Sci.

[163] Cha DH, Roh GH, Hesler SP, Wallingford A, Stockton DG, Park SK, Loeb GM (2021). 2-Pentylfuran: a novel repellent of *Drosophila suzukii*. Pest Manag Sci.

[164] Wood WF, Farquar GR, Largent DL (2000). Different volatile compounds from mycelium and sporocarp of *Pleurotus ostreatus*. Biochem Syst Ecol.

[165] Hofstetter RW, Chen Z, Gaylord ML, McMillin JD, Wagner MR (2008). Synergistic effects of α-pinene and exo-brevicomin on pine bark beetles and associated insects in Arizona. J Appl Entomol.

[166] Haselton AT, Acevedo A, Kuruvilla J, Werner E, Kiernan J, Dhar P (2015). Repellency of α-pinene against the house fly, *Musca domestica*. Phytochemistry.

[167] Kaplan I (2012). Attracting carnivorous arthropods with plant volatiles: the future of biocontrol or playing with fire. Biol Control.

[168] Pattrick JG, Shepherd T, Hoppitt W, Plowman NS, Willmer P (2017). A dual function for 4-methoxybenzaldehyde in *Petasites fragrans*? Pollinator-attractant and ant-repellent. Arthropod Plant Interact.

